# Do Manual and Voxel-Based Morphometry Measure the Same? A Proof of Concept Study

**DOI:** 10.3389/fpsyt.2014.00039

**Published:** 2014-04-08

**Authors:** Niels K. Focke, Sarah Trost, Walter Paulus, Peter Falkai, Oliver Gruber

**Affiliations:** ^1^Department of Neurology/Epileptology and Hertie Institute of Clinical Brain Research, University Medical Center, Eberhard Karls University, Tuebingen, Germany; ^2^Department of Clinical Neurophysiology, University Medical Center, Georg August University, Goettingen, Germany; ^3^Department of Psychiatry and Psychotherapy, Center for Translational Research in Systems Neuroscience and Clinical Psychiatry, Georg August University, Goettingen, Germany; ^4^Department of Psychiatry and Psychotherapy, Ludwig-Maximilians-University, Munich, Germany

**Keywords:** voxel-based morphometry, manual volumetry, validation, DARTEL, mixed psychiatric sample

## Abstract

Voxel-based morphometry (VBM) is a commonly used method to study volumetric variations on a whole brain basis. However, it is often criticized for potential confounds, mainly based on imperfect spatial registration. We therefore aimed to evaluate if VBM and “gold standard” manual volumetry are measuring the same effects with respect to subcortical gray matter volumes. Manual regions-of-interest were drawn in the hippocampus, amygdala, nucleus accumbens, thalamus, putamen, pallidum, and caudate nucleus bilaterally. Resulting volumes were used for a whole brain VBM correlation analysis with Statistical Parametric Mapping (SPM8). The hippocampus, amygdala, putamen, and caudate nucleus were correctly identified by SPM using the contemporary high-dimensional normalization (DARTEL toolbox). This strongly suggests that VBM and manual volumetry both are indeed measuring the same effects with regard to subcortical brain structures.

## Introduction

Since its first description in the late 1990s (1, 2) voxel-based morphometry (VBM) has gained much attention in the neuroscience community and has been applied to pathological and physiological conditions alike. However, from the very beginning there was a general discussion about the validity of the underlying spatial normalization process with the notion that anatomical localization could not be trusted (3). It was hypothesized that global volumetric alterations can be mistaken for local effects. There are several reports in the literature that VBM findings could not be replicated by repeat studies or manual validation, e.g., in schizophrenia (4), which can be regarded as lack of robustness of the method. In the last years, however, revised segmentation (5) and spatial normalization techniques (6) have been described that have improved registration accuracy and thus statistical power (7, 8). To measure volumetric alterations, manual region of interest (ROI)-based methods are still regarded as gold standard by many authors but these are much more time-consuming, subject to operator biased, and require *a priori* anatomical constraints. So far systematic comparisons of automated VBM and manual ROI-based methods have shown conflicting results. One study has reported a superiority of ROI volumetry in physiological aging with an overestimation of age-related differences in regional brain volumes by VBM (9); another study found VBM to be equally specific in detecting local volumetric alterations in expected regions but also capable of detecting remote volume loss in Huntington disease patients (10).

Although VBM- and ROI-based methods are, in principle, measuring similar effects (usually gray volume alterations), the underlying principle is quite different. In the present study, we aimed to investigate whether ROI-based manual volumetry of subcortical brain structures and contemporary VBM in SPM8 (DARTEL toolbox) provide directly correlated results and if these results show anatomical specificity in a large sample of psychiatric patients and healthy controls recruited for different, in part diagnosis-specific, projects of our group. To this end, we obtained manual ROI-derived absolute volumes of subcortical gray matter structures and used these as main effect regressors in a VBM analysis. It was expected that VBM would show significant correlations anatomically associated to the subcortical structure in question.

## Materials and Methods

One hundred and sixty-three subjects participated in the study. The study sample comprised healthy controls (*N* = 54) and psychiatric patients diagnosed with schizophrenia (*N* = 41), bipolar affective disorder (*N* = 41), or obsessive–compulsive disorder (*N* = 27). The mean age was 36.9 ± 12.1 years (range 16–65), 80 subjects were female (see Table 1). The groups were not matched for age and gender; therefore these parameters were included into the voxel-based analysis as covariates of no interest. All subjects gave informed consent and the investigations were approved by the local ethics committee.

**Table 1:** **Demographic and clinical data of study subjects**.

	All subjects (*n* = 163)	Healthy controls (*n* = 54)	Schizophrenia patients (*n* = 41)	Bipolar patients (*n* = 41)	OCD patients (*n* = 27)
Age (years)	36.9 ± 12.1	39.6 ± 12.3	28.4 ± 7.1	43.2 ± 12.2	35.1 ± 9.8
Gender (M/F)	83/80	21/33	28/13	21/20	13/14
Years of education	13.6 ± 2.8	15.4 ± 2.7	12.7 ± 2.7	13.9 ± 2.9	12.7 ± 2.0
Duration of illness in years			0.7 ± 1.2	13.9 ± 10.6	13.7 ± 9.6
MADRS			12.3 ± 8.2	4.6 ± 3.5	9.7 ± 7.3
CGI			4.1 ± 0.9	3.4 ± 1.7	3.9 ± 1.2
BDI			14.5 ± 8.7	7.6 ± 10.4	13.0 ± 10.4
PANSS			88.9 ± 27.7		
YMRS				2.5 ± 2.8	
Y-BOCS					21.0 ± 9.1
CPZ dose equivalents			320.5 ± 303.4	244.7 ± 425.3	

*M/F, male/female; MADRS, Montgomery–Asberg Depression Rating Scale; CGI, clinical global impression; BDI, Beck Depression Inventory; PANSS, Positive and Negative Syndrome Scale; YMRS, Young Mania Rating Scale; Y-BOCS, Yale–Brown Obsessive–Compulsive Scale; CPZ, chlorpromazine dose equivalent (daily), ±SD*.

Structural magnetic resonance imaging (MRI) was carried out using a 1.5 T scanner (Siemens Sonata, Erlangen, Germany). A T1-weighted MPRAGE sequence (TE = 4.42 ms, TR = 1900 ms, TI = 700 ms, flip angle = 15°, FOV 256 mm × 256 mm) of 176 consecutive slices was acquired with a voxel size of 1 mm × 1 mm × 1 mm. Manual ROIs were drawn using the software packages Analyze (1999; Mayo Foundation, Rochester, MN, USA), MRIcro (http://www.cabiatl.com/mricro/) as well as in-house IDL applications as previously described (11–14). First, the magnetic resonance images were realigned in parallel to the anterior commissure–posterior commissure plane. Trained single operators, blinded to the diagnosis, drew outlines of the ROI. These outlines were evaluated for accuracy in the perpendicular cutplanes. The ROI volumes were determined using automatic algorithms programed in MATLAB. ROIs were drawn for the hippocampus, amygdala, nucleus accumbens, thalamus, putamen, pallidum, and caudate nucleus separately for both sides. ROI delineation was done step by step in subsamples by trained single operators. All ROI analyses were initially realized in subsamples over a recruitment period of 4 years in order to answer other scientific, in part diagnosis-specific, questions in the context of different projects of our group. As recruitment was continued after some subsamples had been analyzed by manual morphometry, ROI data was not available for all MRI data sets. The current approach has the advantage that manual volumetry was performed by only one single, trained operator for each ROI without any bias by adding *post hoc* ROI data. Details of the available ROIs per group are given in Table 2.

**Table 2:** **Available ROI volumes per region and group**.

	SZ	BPD	OCD	Controls	All
Hippocampus	41	38	26	53	158
Amygdala	39	41	8	54	142
Accumbens	0	16	17	18	51
Caudate	0	16	17	18	51
Pallidum	0	16	17	18	51
Putamen	0	16	17	18	51
Thalamus	0	16	17	18	51

*SZ, schizophrenia patients; BPD, bipolar affective disorder patients; OCD, obsessive–compulsive disorder patients*.

### Protocol for region of interest delineation

Caudate nucleus (caput): ROIs were drawn on the coronal sections including all gray matter voxels. The tail was not included since this encompasses only very few voxels and is difficult to trace unambiguously. Putamen: this region was drawn on the axial sections with a lateral border at the external capsule, the anterior–medial border at the internal capsule and the posterior–medial border defined by the white matter voxels between putamen and pallidum. Pallidum: this region was also drawn on the axial sections and included all gray matter voxels between the putamen as lateral border and the internal capsule as medial border. The ROI included the lateral and medial parts of the structure as a whole. Nucleus accumbens: this region was drawn on the coronal sections. It was delimited by the inferior border of the head of the caudate nucleus, the internal capsule, and the anterior–medial border of the putamen. Thalamus: this structure was outlined as a whole on the coronal sections and was defined by the posterior aspects of the internal capsule and the third and lateral ventricles as medial and posterior margins, see also Radenbach et al. (13). Hippocampus: this region was drawn on sagittal sections and checked in the other coronal and horizontal views, see Pajonk et al. (11). Amygdala: this region was drawn in the coronal sections. The anterior border was defined by the point when the amygdala became too diffuse to be resolved from the temporo-polar cortex. The superior and lateral borders were defined by the temporal lobe white matter and the inferior border by the white matter of the parahippocampal gyrus as previously described (12, 14, 15).

### Voxel-based analysis

**Figure 1:**
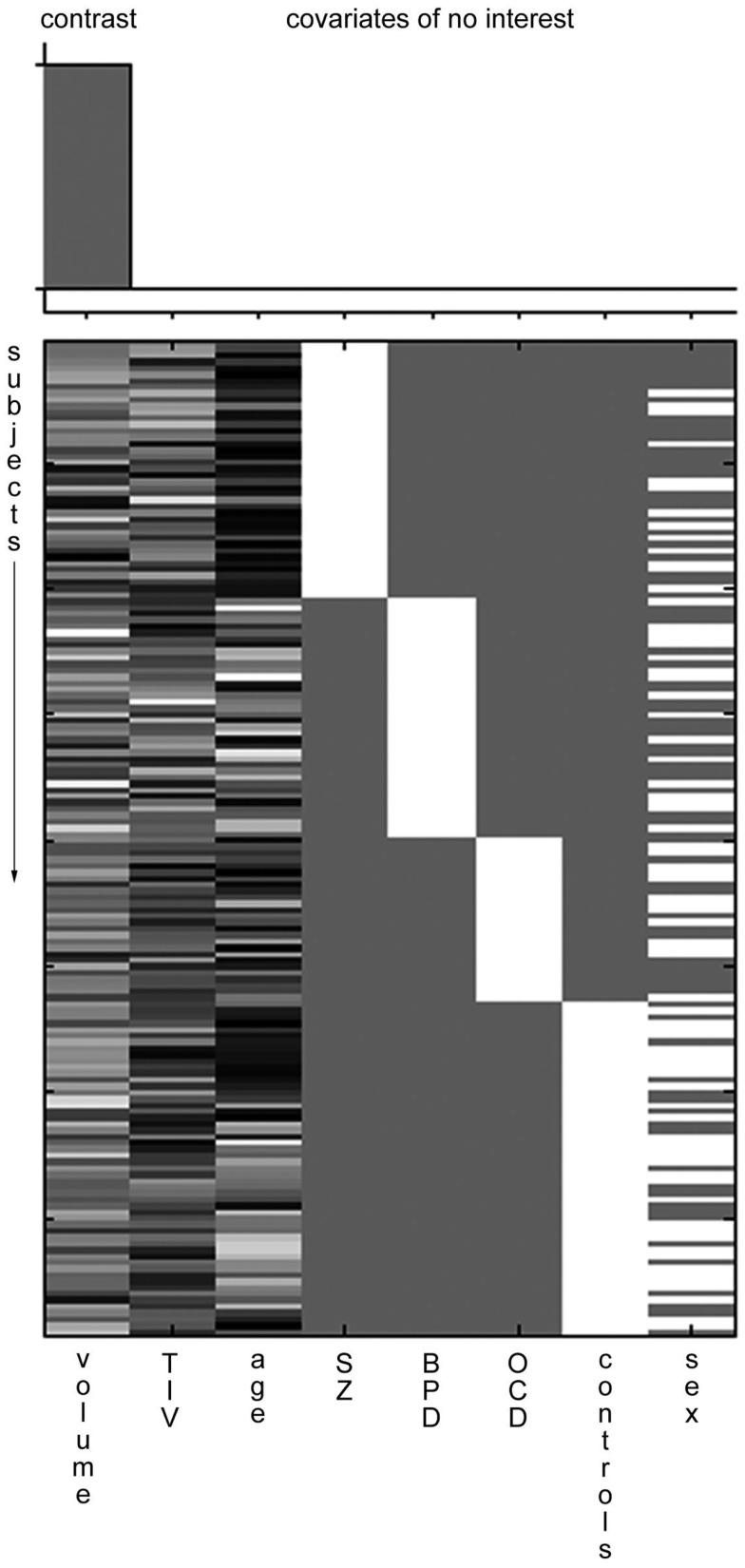
**Example SPM design matrix**. A typical SPM design matrix is shown. The volume column is the main effect contrast, total intracranial volume (TIV), age, diagnostic group (SZ, schizophrenia; BPD, bipolar affective disorder; OCD, obsessive compulsive disorder), and sex are included as covariates of no interest.

Images were converted to NIFTI format and processed on an offline Linux workstation using SPM8. The images were segmented into gray and white matter tissue classes and spatially normalized according to the SPM8 DARTEL procedure with default settings in 1.5 mm cubic resolution and MNI space using a custom, sample-derived template (6). The normalized gray matter maps were modulated with the resulting Jacobian determinant maps and smoothed with an 8-mm FWHM Gaussian kernel. Total intracranial volume (TIV) was estimated by adding up the native space volumes of the gray matter, white matter, and CSF maps in MATLAB. The GLM analysis was in turn set up for each ROI in a multiple regressions design with the absolute, manually measured ROI volume as main effect and including diagnosis group, age, gender, and TIV as covariates of no interest (Figure 1). One-tailed *t* contrasts were then generated using family wise error rate (FWE) correction with a *p* < 0.05 and, additionally (as exploratory test), with an uncorrected *p* < 0.0001 threshold.

## Results

There was a strong positive and significant right–left correlation between all manually drawn ROI volumes (Pearson correlation coefficients were calculated between all right and left subcortical volumes: hippocampus *r* = 0.361, *p* < 0.001; amygdala *r* = 0.813, *p* < 0.001; nucleus accumbens *r* = 0.695, *p* < 0.001; caudate *r* = 0.927, *p* < 0.001; pallidum *r* = 0.687, *p* < 0.001; putamen *r* = 0.938, *p* < 0.001; thalamus *r* = 0.754, *p* < 0.001; all two-sided significant).

**Figure 2:**
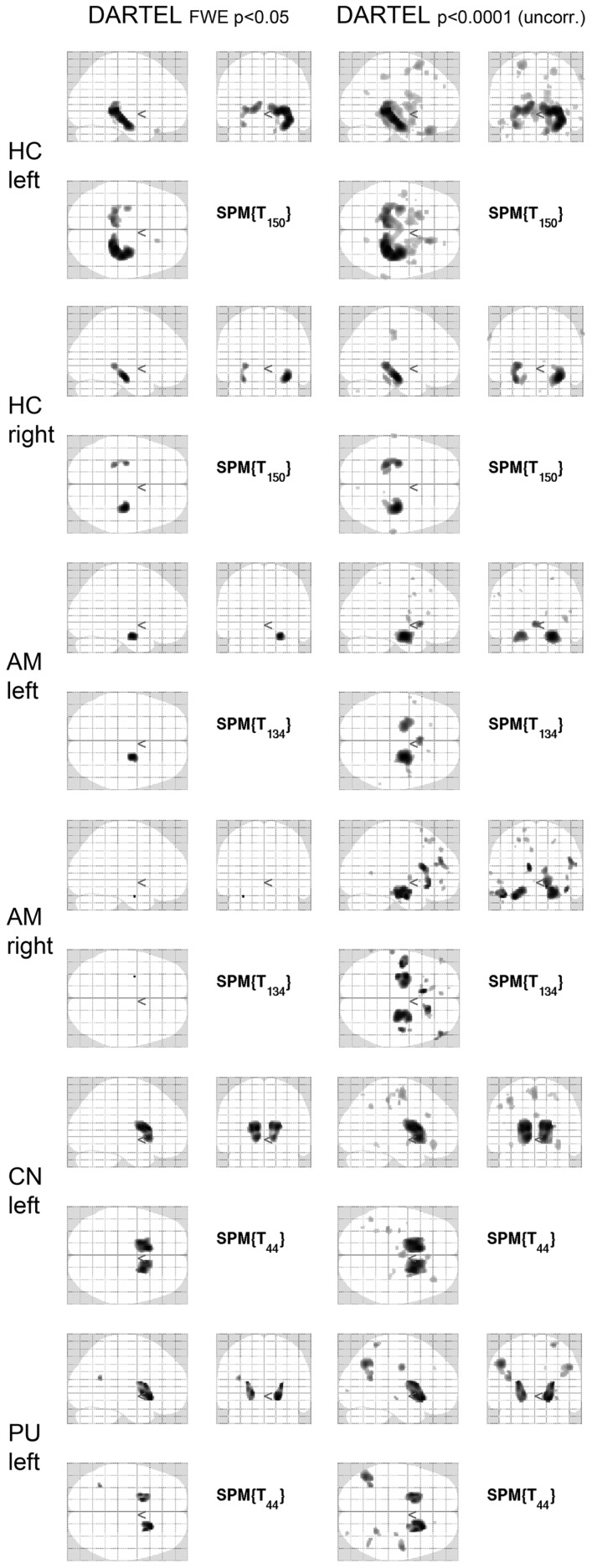
**SPM results in glass brain projection**. SPM glass brains for different regions and thresholds [*p* < 0.05 (FWE corrected) and *p* < 0.0001 (uncorrected)] are shown. Images are in neurological convention (left in the image is left in the subject). FWE, family-wise error correction; DARTEL, normalization done with DARTEL-toobox; HC, hippocampus; AM, amygdala; CN, caudate nucleus; PU, putamen.

**Figure 3:**
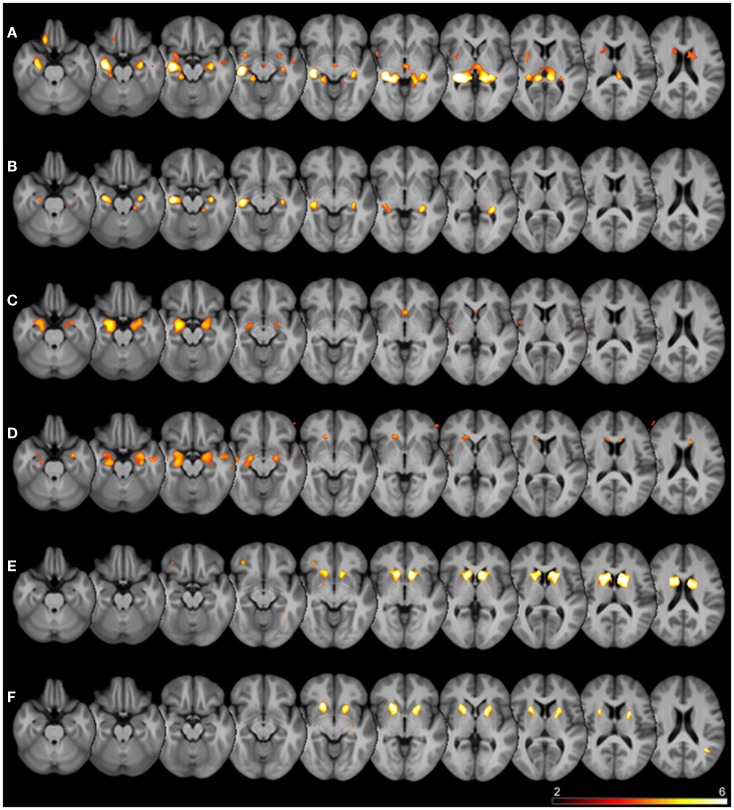
**Superimposed SPM results**. Thresholded SPM results (uncorrected *p* < 0.0001) superimposed on the averaged T1-weighted images of all subjects are shown. The color scale represents SPM t-scores, images are in radiological convention (left in image is right in the subject). Rows are different seed regions: **(A)** left hippocampus, **(B)** right hippocampus, **(C)** left amygdala, **(D)** right amygdala, **(E)** left caudate nucleus, **(F)** left putamen.

Details of the SPM results using DARTEL processing are given in Table 3, overview glass brain images are shown in Figure 2, exemplary axial cutplane images in Figure 3.

**Table 3:** **SPM results (DARTEL)**.

ROI	*k*	*T*	MNI	Anatomical position
Left hippocampus	2173	7.53	26 −30 1	Right hippocampus
		7.39	36 −21 −11	
		7.38	32 −13 −20	
	776	6.24	−14 −36 3	**Left hippocampus**
		5.98	−24 −31 1	
		5.86	−6 −31 10	
		5.59	−30 −15 −18	
	33	5.10	15 28 −27	Right fronto-orbital
	12	4.90	20 −33 −15	Right parahippocampal gyrus
Right hippocampus	466	6.60	33 −18 −15	**Right hippocampus**
	209	5.64	−30 −16 −18	Left hippocampus
		5.50	−26 −33 0	
		4.98	−32 −24 −9	
Left amygdala	306	5.34	22 −7 −18	Right amygdala
	*N/A*	*4.74*	*−20 −6 −18*	***Left amygdala***
	*N/A*	*4.59*	*−2 15 −2*	*Subcallosal cortex*
	N/A	4.08	42 −1 4	Right insular cortex
	N/A	4.06	22 5 60	Right superior frontal gyrus
	N/A	3.99	50 0 15	Right central opercular cortex
	N/A	3.96	−52 8 39	Left middle frontal gyrus
	N/A	3.92	15 27 13	Right caudate
	N/A	3.87	−60 0 36	Left precentral gyrus
	N/A	3.85	−22 30 45	Left superior frontal gyrus
	N/A	3.85	60 −42 54	Right supramarginal gyrus
Right amygdala	2	4.85	−30 −4 −23	Left amygdala
	*N/A*	*4.76*	*24 −13 −18*	***Right amygdala***
	*N/A*	*4.74*	*−9 20 16*	*No gray matter structure (left anterior callosum)*
	*N/A*	*4.72*	*−48 −12 −23*	*Left inferior temporal gyrus*
	*N/A*	*4.67*	*15 27 −9*	*Right fronto-orbital*
	*N/A*	*4.37*	*50 45 27*	*Right frontal pole*
Left caudate	1193	7.60	−9 15 12	**Left caudate**
		7.32	−16 5 13	
		7.05	−9 17 −2	
	883	7.29	20 8 13	Right caudate
		6.91	10 14 15	
		6.82	15 15 0	
Right caudate	1113	9.08	−20 5 13	Left caudate
		7.07	−12 14 13	
		6.27	−14 12 −2	
	1128	9.06	22 8 15	**Right caudate**
		6.74	15 14 0	
Left putamen	413	6.75	20 18 −6	Right putamen
		6.42	24 12 10	
	366	6.49	−16 8 1	**Left putamen**
		6.30	−24 5 15	
		6.10	−22 14 6	
	25	5.88	−34 −54 21	Left angular gyrus
Right putamen	206	6.57	21 21 −8	**Right putamen**
	27	6.22	26 12 12	Right insula
	36	6.13	−24 6 15	Left putamen
	19	5.70	−15 9 −2	Left putamen
	14	5.57	−48 −61 40	Left lateral occipital cortex
	4	5.44	−18 15 −8	Left putamen
Left pallidum	*N/A*	*4.97*	*−26 −10 −9*	*Left amygdala*
	*N/A*	*4.75*	*21 27 −8*	*Frontal orbital cortex*
	*N/A*	*4.66*	*2 −4 −14*	*No label found*
	*N/A*	*4.44*	*33 39 −9*	*Right frontal pole*
	*N/A*	*4.36*	*−38 −28 49*	*Left postcentral gyrus*
	*N/A*	*4.31*	*14 23 −5*	*Right caudate*
Right pallidum	No suprathreshold clusters at *p* < 0.05 (FWE) and *p* < 0.0001 (uncorrected)			
Left thalamus	*N/A*	*4.27*	*−14 54 42*	*Left frontal pole*
	*N/A*	*4.24*	*2 −9 −2*	*Right thalamus*
Right thalamus	*N/A*	*5.28*	*22 24 −8*	*Right putamen*
	*N/A*	*5.26*	*−28 32 34*	*Left middle frontal gyrus*
	*N/A*	*5.01*	*38 33 −9*	*Right frontal orbital cortex*
	*N/A*	*4.88*	*−15 50 49*	*Left frontal pole*
	*N/A*	*4.78*	*−44 −57 28*	*Left angular gyrus*
	*N/A*	*4.55*	*10 0 −2*	*Right pallidum*
	*N/A*	*4.42*	*12 48 18*	*Right paracingulate gyrus*
	*N/A*	*4.41*	*15 32 39*	*Right frontal pole*
	*N/A*	*4.38*	*−64 −31 22*	*Left supramarginal gyrus*
	*N/A*	*4.36*	*−44 3 12*	*Left central opercular cortex*
Left accumbens	No suprathreshold clusters at *p* < 0.05 (FWE) and *p* < 0.0001 (uncorrected)			
Right accumbens	*N/A*	*4.35*	*35 −6 66*	*Right precentral gyrus*
	*N/A*	*4.29*	*56 18 −20*	*Right temporal pole*

*All significant clusters/sub-clusters as given by SPM (DARTEL processing stream) at a threshold of *p* < 0.05 (corrected with FWE) are shown. *T*-scores (*T*) are peak level per cluster, local maxima are grouped and ordered according to *T*-score. Lines in italics are from the exploratory test (*p* < 0.0001 uncorrected) and are given when the structure in question did not survive error correction (clusters <10 voxel are not shown for the purpose of clarity). Anatomical position was determined using FSLview atlas tool and the Harvard–Oxford Cortical/Subcortical Structural Atlas. Anatomical position is given in bold when matching with the ROI structure. *k*, Cluster size/number of voxels in the FWE-corrected analysis; MNI, coordinates according to the Montreal Neurological Institute template (SPM8); N/A, not available*.

SPM detected significant correlations (*p* < 0.05 FWE-corrected) of the manually measured ROI volumes and the anatomically corresponding gray matter volume as measured by VBM in both hippocampi, both caudate nuclei, and both putamina. Interestingly, in all these analyses the structure in question was detected bilaterally, sometimes with slightly higher *T*-scores on the contralateral side. For the amygdala ROI, only the respective contralateral structure survived the error correction; however, with the exploratory threshold both amygdalae were detected. For the pallidum and nucleus accumbens ROIs, no suprathreshold clusters could be found both with the conservative FWE-corrected analysis as well as with the uncorrected *p* < 0.0001 threshold. The thalamus ROI volumes also showed no suprathreshold correlations within the structures in question. With an even lower significance threshold of *p* < 0.001 (uncorrected), the left pallidum and both thalami could be identified, but with this threshold 20 (left pallidum), 19 (left thalamus), or 46 (right thalamus) suprathreshold clusters outside the ROI would also be detected (data not shown).

## Discussion

In this study, we assessed whether voxel-based morphometric analyses using SPM8 (DARTEL) and manual volumetry would show results consistent with each other in a mixed sample of patients with schizophrenia, bipolar affective disorder, and obsessive–compulsive disorder and healthy controls. In particular, we performed GLM analyses by entering the manually determined volumes of different ROIs in a (multiple) regression design. From a theoretical point of view, the observed variations of the ROI-measured subcortical volumes should be closely correlated to the VBM-measured local gray matter volume in the corresponding voxels. This design, thus, allowed for clear *a priori* hypotheses where significant correlations should be localized.

In fact, these hypotheses were confirmed in the DARTEL processing stream for both hippocampi, amygdalae, caudate nuclei, and putamina, but not for the nucleus accumbens, thalamus, and pallidum. These would have required even lower significance thresholds (*p* < 0.001 and less) that are usually not acceptable in a whole brain analysis. Several possible explanations may account for the partially negative results in these regions. The nucleus accumbens is difficult to outline in manual volumetry due to proximity to the caudate nucleus and putamen. Thalamus and pallidum on the other hand are structures that are not homogenously segmented as gray matter by SPM: the thalamus is divided into multiple subnuclei with intersecting white matter tracts, whereas the manual ROI tracing was done following the outer boundaries of the structure as a whole. The pallidum is iron rich causing susceptibility effects and automated standard methods based on T1-weighted images often fail to segment this structure correctly (16). Also for these regions, the available *N* was lower in comparison to the hippocampus and amygdala. Nevertheless, as the same *N* was sufficient to show strong correlations in the caudate and putamen, it is unlikely that this effect was purely power-dependent. Another interesting finding is the strong bilaterality of correlations in the VBM results. As the manually measured volumes were also strongly right–left correlated, the contralateral structure in question was always detected in the VBM analysis as well, sometimes even with slightly higher significance levels. This highlights that, in a mixed sample of psychiatric patients and controls, volume alterations are not strongly lateralized. This is in keeping with a large meta-analysis of hippocampus volumes in schizophrenic patients that found a highly significant volume loss without any side preference (4). Also in depression, amygdala volumes were affected bilaterally although the direction of alterations seems to be influenced by drug effects (17). Another MRI study reported reduced thalamic volumes in major depressive disorder, which was also symmetric (18).

With a clear hypothesis or with an exploratory intention, it can be useful and justified to apply a more liberal significance threshold, e.g., *p* < 0.0001 (uncorrected). In our sample, this was necessary to detect the ipsilateral amygdala that would not have survived full-brain FWE correction. This approach did, however, impact on specificity as additional clusters occurred outside the principal ROI (Table 3). Of note, covariance in homotopic, but also ipsilateral and heterotopic gray matter densities measured by VBM has been reported (19) and is seen, for example, in age-related decline (20). Therefore, these additional clusters may be explained by structural covariance, although it cannot be excluded that some are spurious. In summary, we could demonstrate that VBM, particularly the contemporary DARTEL-based variant, is in fact measuring the same effect as manual volumetry in most subcortical regions and shows high anatomical specificity. Further studies, however, are needed to evaluate the impact of VBM for cortical regions.

## Author Contributions

All authors and co-authors contributed substantially to this work. Niels K. Focke, Oliver Gruber, Peter Falkai, and Walter Paulus initiated and designed the study. Oliver Gruber and Peter Falkai were involved in data acquisition. Niels K. Focke, Oliver Gruber, and Sarah Trost analyzed and interpreted the data. Niels K. Focke, Oliver Gruber, and Sarah Trost critically discussed the results. Niels K. Focke and Sarah Trost wrote the manuscript. Oliver Gruber, Peter Falkai, and Walter Paulus critically revised the manuscript. Sarah Trost finalized the manuscript. All authors and co-authors approved the final version of the manuscript.

## Conflict of Interest Statement

The authors declare that the research was conducted in the absence of any commercial or financial relationships that could be construed as a potential conflict of interest.
